# Combination of *dl922-947* Oncolytic Adenovirus and G-Quadruplex Binders Uncovers Improved Antitumor Activity in Breast Cancer

**DOI:** 10.3390/cells11162482

**Published:** 2022-08-10

**Authors:** Fabiana Napolitano, Sarah Di Somma, Giuliano Castellano, Jussara Amato, Bruno Pagano, Antonio Randazzo, Giuseppe Portella, Anna Maria Malfitano

**Affiliations:** 1 Department of Translational Medical Sciences, University of Naples Federico II, 80131 Naples, Italy; 2 Department of Pharmacy, University of Naples Federico II, 80131 Naples, Italy

**Keywords:** oncolytic virus, adenovirus, G-quadruplex, breast cancer, senescence, STING

## Abstract

G-quadruplexes (G4s) are nucleic secondary structures characterized by G-tetrads. G4 motif stabilization induces DNA damage and cancer cell death; therefore, G4-targeting small molecules are the focus of clinical investigation. DNA destabilization induced by G4 ligands might potentiate the anticancer activity of agents targeting DNA or inhibiting its repair such as oncolytic viruses. This study represents the first approach combining G4 ligands, BRACO-19 (B19), pyridostatin (PDS), and the adenovirus *dl922-947* in breast cancer cells. We demonstrated that G4 binders and *dl922-947* induce cytotoxicity in breast cancer cells (MDA-MB-231 and MCF-7) and at higher doses in other neoplastic cell lines of thyroid (BHT-101 cells) and prostate (PC3 cells). G4 binders induce G4 motifs distributed in the S and G2/M phases in MCF-7 cells. G4 binder/*dl922-947* combination increases cell cytotoxicity and the accumulation in subG0/G1. Indeed, G4 binders favor viral entry and replication with no effect on coxsackie and adenovirus receptor. Notably, *dl922-947* induces G4 motifs and its combination with PDS potentiates this effect in MCF-7 cells. The agents alone or in combination similarly enhanced cell senescence. Additionally, PDS/*dl922-947* combination inactivates STING signaling in MDA-MB-231 cells. Our results suggest that G4 binder/virotherapy combination may represent a novel therapeutic anticancer approach.

## 1. Introduction

In recent years, the identification of novel therapeutic targets has led to the development of more selective and active drugs against neoplastic lesions; however, some lesions with aggressive features and poor prognosis still lack effective treatments, addressing the research to evaluate novel therapeutic combinations. Studies suggest the anticancer potential of small molecules selectively targeting nucleic acid G-quadruplex (G4) motifs [1,2]. G4 secondary structures are characterized by stacking of planar cyclic G-tetrads in which four guanine bases are connected by Hoogsteen hydrogen bonds [3]. The distribution of G4-forming sequences in the human genome is detectable at the telomeres and promoter regions of several proto-oncogenes [4]. The anticancer potential of G4 binders able to stabilize G4 motifs has been investigated in tumor cell lines and the data obtained warrant further evaluation in clinical trials [5].

The anticancer activities of G4 binders include cell cycle arrest, DNA damage, induction of senescence, and apoptosis [6]. We recently demonstrated the anticancer effects in prostate and breast cancer cell lines of two G4-targeting ligands endowed with high affinity and good selectivity toward telomeric G4 motifs, BRACO-19 (B19), and C066-3108 (C066) [7]. In the triple negative MDA-MB-231 aggressive breast cancer cell line, high DNA damage was associated to induction of immunogenic cell death (ICD) and subsequent human T cell activation [7]. In ER+ MCF-7 breast cancer cell line with characteristics of differentiated mammary epithelium, both compounds induced only a modest DNA damage associated to apoptosis induction.

We hypothesized that G4 motif formation and stabilization in cancer cells might be suitable to potentiate the antitumor activity of other agents activating the DNA damage repair (DDR) system, such as the oncolytic viruses (OVs). The use of OVs represents a promising therapeutic option for lesions with poor prognosis and virotherapy-based approaches have gained successful outcomes in several clinical setting [8]. OV anticancer activity is prompted by a direct lytic effect on cancer cells; indirect effects have also been observed such as the block of DDR with subsequent activation of cell death pathways. Other indirect effects include the induction of innate and adaptive anti-tumor immune response with a re-shape of the tumor microenvironment (TME) towards an antitumor phenotype [9,10], the release of cytokines, tumor-associated antigens (TAAs), and damage-associated and pathogen-associated molecular patterns (DAMPs, PAMPs), able to induce ICD [11,12]. The oncolytic *dl922-947* adenovirus demonstrated potent anticancer effects both in vitro and in vivo in breast cancer models [13]. This adenoviral mutant bears a 24 bp deletion in the E1A-Conserved Region 2 (CR-2), therefore the mutant virus can replicate only in cells with a non-functional retinoblastoma (RB) pathway, a defect observed in almost all the human malignancies [14]. We proved the anticancer efficacy of *dl922-947* as a single agent or in combination with other agents in experimental models [15,16,17,18,19] describing several mechanisms leading to cancer cell death. We observed that *dl922-947* impairs the DDR system and its effect is potentiated by ionizing radiation, ATM, and PARP inhibitors [20]. We observed cell growth and cycle arrest accompanied by ICD in malignant mesothelioma cells with a potentiated cytotoxic effect with chemotherapeutic drugs in vitro [7]. In the same cell models, the combination of *dl922-947* with inhibitor of the G2/M DNA damage checkpoint kinase WEE1 elicited a synergic induction of cell death and DDR [15,16].

We hypothesized that the specific structural changes induced in DNA of breast cancer cells by G4 ligands might potentiate the cell death pathways (DDR and ICD) known to be stimulated by *dl922-947* [11,16]. Indeed, combinatory effects of *dl922-947* with G4 binders are completely unexplored. Among G4 binders, we used B19 and pyridostatin (PDS) in this study, both reported to exert anticancer activity in breast cancer cells; in particular, PDS reduced Src protein levels and Src-dependent cell motility [7,21].

## 2. Materials and Methods

### 2.1. Cells and Adenovirus

MCF-7 and MDA-MB-231 breast cancer cell lines, PC3 prostate cancer cell line, and BHT-101 anaplastic thyroid carcinoma cell line were cultured in DMEM (GIBCO, Paisley, UK) supplemented with 2 mM L-glutamine, 50 ng/mL streptomycin, 50 units/mL penicillin, and 10% heat-inactivated fetal bovine serum (FBS) (GIBCO). MCF-10A cells were cultured in DMEM-1640 10% FBS supplemented with 2 mM L-glutamine, 50 ng/mL, streptomycin, 50 units/mL penicillin, epithelial growth factor (EGF) 40 ng/mL, and insulin 5 µg/mL. Cells purchased from ATCC were maintained in a humidified atmosphere with 5% CO_2_ at 37 °C. Cells reaching 70–80% of confluence were harvested with 0.25% trypsin (Sigma-Aldrich, St Louis, MO, USA) and used for the experiments. *dl922-947* and *Ad*GFP viral stocks were expanded in the human embryonic kidney cell line HEK-293, purified, stored, and quantified (1.22 × 10^8^ p.f.u./mL) as previously reported [22].

### 2.2. Sulforhodamine B (SRB) Cytotoxicity Assays

MCF-7 (500 cells/well), MDA-MB-231 (500 cells/well), BHT-101 (500 cells/well), PC3 (1000 cells/well), MCF-10A (100 cells/well) cells were seeded in triplicates in 96-well plates and allowed to adhere for 24 h. G4 ligands, B19 and PDS (where indicated), were added at increasing concentrations to the cell cultures (from 0.1 to 10 µM). *dl922-947* was added to MDA-MB-231 and MCF-7 cell cultures in a range from 0.1 to 10 p.f.u. × cell (not shown). After 6 days of incubation, cells were fixed with 50% *v*/*v* trichloroacetic acid for 2 h under stirring at 4 °C. Cells were washed with distillated water, dried overnight, and stained with 0.4% *w*/*v* SRB in 1% *v*/*v* acetic acid at room temperature for 30 min on shaker. Washes were made with 1% acetic acid until the removal of the unbound dye and the plates were left to dry. The dye was solubilized in TRIS-HCl 10 mM as previously described [23,24]. The reading was performed at 495 nm by Glomax^®^ Discover Microplate Reader (Promega, Madison, WI, USA). The doses of *dl922-947* or G4 ligands required to inhibit 50% of cell viability (half maximal inhibitory concentration, IC_50_) and/or 25% of cell viability IC_25_) were determined by GraphPad Prism 7 Software, San Diego, California, USA.

### 2.3. G4 Motif Formation and Cell Cycle Analyses

MCF-7 and MDA-MB-231 cell lines were cultured in 24-well plates (8000 cells/well) in the presence and absence of G4 ligands or *dl922-947* at IC_50_ doses for six days. The combination *dl922-947*/G4 binders was also settled. After the incubation, cells were detached with trypsin, washed twice with PBS, fixed in 70% (*v*/*v*) ethanol, and stored at −20 °C at least overnight for the determination of the cell cycle [25,26] and G4 motifs [7]. The cell pellet, washed with PBS/Tween buffer (PBT) (0.5% *w*/*v* BSA and 0.1% *v*/*v* Tween 20 in PBS), was re-suspended in PBT and stained with BG4 monoclonal antibody (Millipore, Burlington, MA, USA, MABE 917) for 1 h at room temperature. Samples were washed with PBT and stained with Alexa Fluor 488 anti-mouse (Invitrogen, Waltham, MA, USA, #A11001, 1:100) in PBT at darkness. After 30 min, cells were washed with PBT and re-suspended in PBS containing RNaseA (Roche) (0.4 U) and PI (0.015 mol/L). Flow cytometry acquisition was performed for the emission in FL1 and FL3 channels for the determination of G4 structures and in the FL3 channel for the acquisition of the cell cycle with a BD LSRFortessa (BD Biosciences, San Jose, CA, USA). The analyses were performed using Flowlogic Software (MACS, Milteny Biotech, Bergisch Gladbach, North Rhine-Westphalia, Germany). To remove artifacts, doublets, and aggregates, an electronic doublet discrimination was performed using the area versus width of the fluorescence (FL3) pulse.

### 2.4. AdGFP Infection

MCF-7 and MDA-MB-231 cells were seeded (12,500 cells/well) in 24-well-plates and after 24 or 48 h infected with *Ad*GFP (30 p.f.u/cell). G4 binders were added at their IC_50_ values simultaneously (24 h after the adherence of the cells to the plastic) with the virus, or 24 h after virus infection, or 24 h before virus infection. After 48 h of incubation, cells were detached with trypsin and the pellet was re-suspended in PBS to read GFP emission by flow cytometry.

### 2.5. Evaluation of *dl922-947* DNA Amplification

MCF-7 and MDA-MB-231 cells (10,000 cells/well) were seeded in duplicate in 12-well plates and 24 h later were infected with *dl922-947* at the IC_50_ value (or *Ad*GFP at 30 p.f.u. × cell, used as control) and treated with G4 ligands at their IC_50_ values. Cells and supernatants were separately collected 24 and 48 h post infection (hpi). Cell pellets were disrupted by three freeze–thaw cycles to favor the release of the virus and centrifuged at 1000 g for 5 min, the supernatants were collected. Viral DNA was extracted from supernatants and cell pellets by High Pure Viral Nucleic Acid Kit (Roche, Basel, Switzerland) and quantified by qPCR using the primers for viral HEXON gene expression.

### 2.6. Induction of Cell Senescence

MDA-MB-231 (30,000 cells/well) and MCF-7 (40,000 cells/well) cells were seeded in 6-well plates and 24 h later were infected with *dl922-947* at the IC_50_ value or treated with G4 ligands. Virus/G4 ligand combinations were also examined. After 72 h, we assessed the senescence using the SA-b-gal Staining Kit (Cell BioLabs, San Diego, CA, USA). Cells were washed twice with PBS (1×) and incubated at room temperature for 5 min with 1× Fixing Solution. Cells were washed three times with PBS, the final wash was aspirated, and the cells were completely covered with freshly prepared Cell Staining Working Solution in the dark for 4 h at 37 °C. After the incubation, the solution was removed, the cells were washed twice with PBS and blue-stained senescence cells were observed by a light microscope and counted at 490 nm by Glomax^®^ Discover Microplate Reader (Promega, Madison, WI, USA).

### 2.7. CAR Modualtion by G4 Binders and STING Signaling Modulation by G4 Binders/dl922-947

To address CAR expression, MCF-7 (3 × 10^5^ cells) and MDA-MB-231 (3 × 10^5^ cells) cells were treated with B19 and PDS at the IC_50_ for 48 h. To analyze STING signaling, MCF-7 (4 × 10^5^ cells) and MDA-MB-231 (4 × 10^5^ cells) cells were treated with B19, PDS, and *dl922-947* and their combinations at IC_50_ doses for 24 h. After the treatments, cells were lysed in R.I.P.A. buffer (50 mMTris-HCl, pH = 7.4; 150 mM NaCl; 0.5 M EDTA, 1% NP-40; 0.5% sodium deoxycholate; 0.1% SDS; 1:100 phosphatase and protease inhibitors) and centrifuged (17,000 g for 20′ at 4 °C). Protein concentration was determined by Bradford Protein Assay (Bio-Rad, Berkeley, CA, USA), extracts (30 µg) were diluted in Laemmli sample buffer and subjected to SDS-PAGE. Samples were resolved under constant voltage (100 mA) and transferred to PVDF membranes (Millipore Corporation, Darmstadt, Germany). Blots were blocked with 5% BSA in TBS containing 0.1% Tween-20 for 1 h at room temperature. Filters were incubated overnight at 4 °C using the following antibodies: anti-STING (MA5-26026, 1:1000, Invitrogen, Rockford, IL, USA) and anti-α-tubulin (T9026, 1:10,000, SIGMA, Saint Louis, MO, USA). Blots were incubated for 1 h with horseradish peroxidase conjugated goat anti-mouse IgG (Biorad, Berkeley, CA, USA) and then revealed by an enhanced chemiluminescence (ECL) system (Thermo Scientific, Rockford, IL, USA) as previously described [27,28]. Immunoreactive bands were quantified by Image j (Bethesda, MD, USA) Bethesda, USA software.

### 2.8. Statistical Analysis

Statistical analysis was performed by Prism 7 (GraphPad Software). Statistical significance of differences among groups was analyzed by ANOVA corrected for multiple comparisons) or by parametric paired two tailed t test (as indicated in the figure legends) and p values ≤ 0.05 were considered statistically significant.

## 3. Results

### 3.1. Anti-Proliferative Effects of B19, PDS, and dl922-947

We compared the sensitivity of different cancer cell lines to B19-induced cytotoxicity performing dose-response proliferation assays and evaluating the IC_50_ values. We observed that MDA-MB-231 and MCF-7 cells elicited a lower IC_50_ with respect to the other cell lines used, anaplastic thyroid cancer cells, BHT-101, and prostate cancer cells, PC3 (Table 1A). Our data confirm the sensitivity of these cell lines to B19 as previously demonstrated in another study [7]. Additionally, we tested B19 in MCF-10A cells to exclude cytotoxic effects in a non-cancerous breast epithelial cell line. We observed no inhibition of cell proliferation as assessed by SRB assays (Figure) as previously reported [7]. Breast cancer cell lines were selected for further assays. We compared the effect of B19 to PDS showing dose-dependent inhibition of cell viability. We observed that B19 and PDS achieved a similar IC_50_ value in MDA-MB-231 cells, whereas the values were higher in MCF-7 cells, particularly with B19 (Table 1B). The difference of IC_50_ values between the two cell lines might indicate a different sensitivity to the treatment with G4 binders. A previous study used oncolytic adenoviruses in breast cancer cells and reported higher cytotoxic activity of *dl922-947* with respect to other adenoviruses [13]. In agreement with this study, we observed a dose-dependent cytotoxic effect of *dl922-947* in MDA-MB-231 and MCF-7 cell lines as demonstrated by the IC_25_ and IC_50_ values reported in Table 1B.

### 3.2. G4 Motifs in Breast Cancer Cells

**Figure 1:**
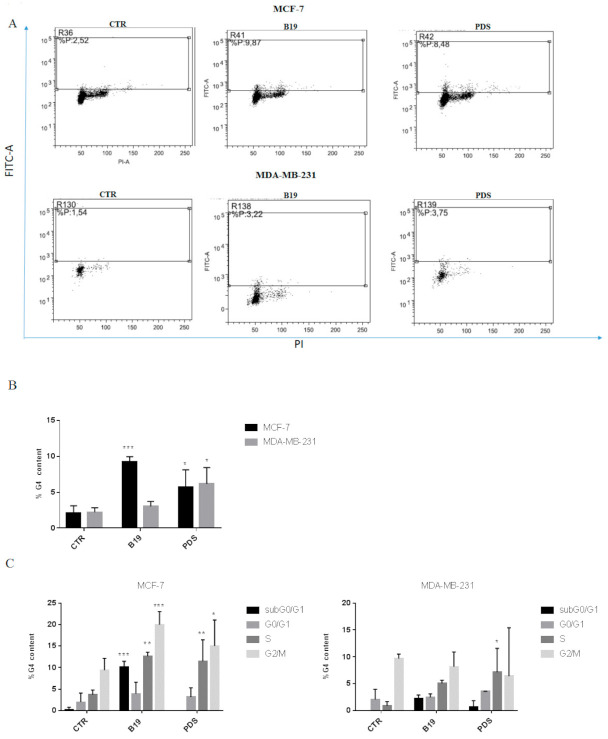
G4 motif content and distribution in cell cycle phases. (**A**) MDA-MB231 and MCF-7 cells were treated with G4 ligands at the IC_50_ values. The dot plot profiles in the upper fluorescein isothiocyanate (FITC) gated panel indicate the amount of positivity for G4 motif. The *x* axis reports PI positivity to specifically stain cell DNA. In the dot plot reported, the percent of positive cells for each experimental conditions is indicated in the gated region (R) for a single representative experiment, whereas the histograms (**B**) represent the mean ± SD of at least three independent experiments. The statistical analysis was performed by GraphPad Prism 7 by two-way ANOVA using Dunnet’s multiple comparisons test. In the panel (**C**), G4 motif distribution is reported by evaluation of PI positive cells (PI-W versus PI-H dot plot) in each phase of the cell cycle as the percent of G4 motif positivity. The statistical significance was calculated by GraphPad Prism 7 with two-way ANOVA using Tukey’s multiple comparisons test. The calculated *p* values are: *** *p* < 0.0001, ** *p* < 0.005, * *p* < 0.05.

We investigated G4 motif formation following PDS and B19 treatment (at the IC_50_ values) in MDA-MB-231 and MCF-7 cells. We gated PI positive cells (dot plot PI-H vs. PI-W) and analyzed G4 content as anti-BG4 positive cells (Figure 1A,B). As previously observed, we detected low basal levels of G4 motifs [7]. B19 induced G4 motifs in MCF-7 cells; a slight, although not statistically significant increase was observed in MDA-MB-231 cells. The induction of G4 motifs by PDS was dose-dependent (Figure S2) and statistically significant in both cell lines (Figure 1A,B). G4 motif distribution was analyzed gating sub-regions within anti-BG4 positive cells corresponding to the sub-G0/G1, G0/G1, S, and G2/M phases of the cell cycle (Figure 1C). B19 induced in MCF-7 cells an increase in the subG0/G1, S, and G2/M phases with respect to the control, whereas in MDA-MB-231 cells, G4 motifs were detected in G2/M phase as in the control. PDS increased G4 content in S and G2/M phases in MCF-7 cells, and in S phase in MDA-MB-231 cells (Figure 1C). The presence of G4 motifs in the subG0/G1 phase correlates with induction of apoptosis previously observed treating MCF-7 cells with B19 [7], thus suggesting that in apoptotic cells, G4 motifs seem to be stabilized. The presence of G4 motifs in S and G2/M phases correlates with their formation during cell synthesis and division as reported in a previous study [29].

### 3.3. Cytotoxic Effects of dl922-947/G4 Binder Combination

To address a potential increase of cytotoxicity due to combinatory treatment of G4 binders with *dl922-947*, we used both the agents at the IC25 and IC_50_ added simultaneously to the cell cultures (data not shown). From these assays, we selected the combination of the virus at its IC_50_ dose and G4 binders at the IC_25_ and IC_50_ values.

**Figure 2:**
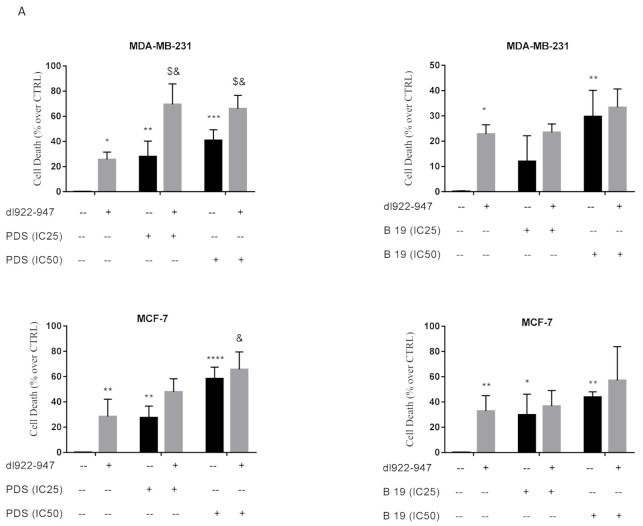

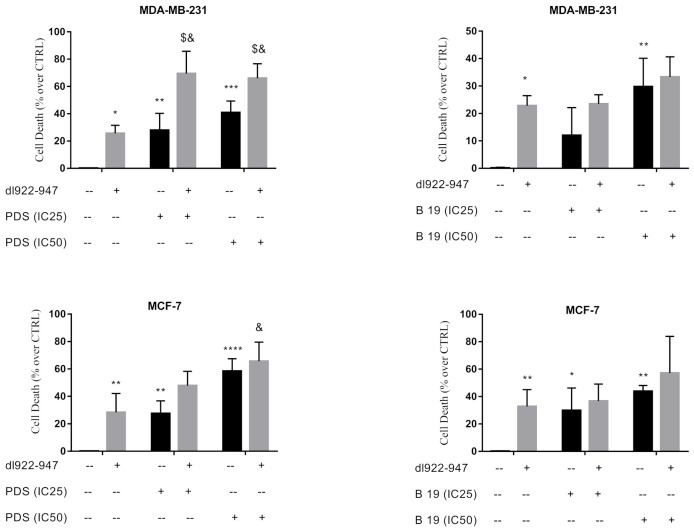

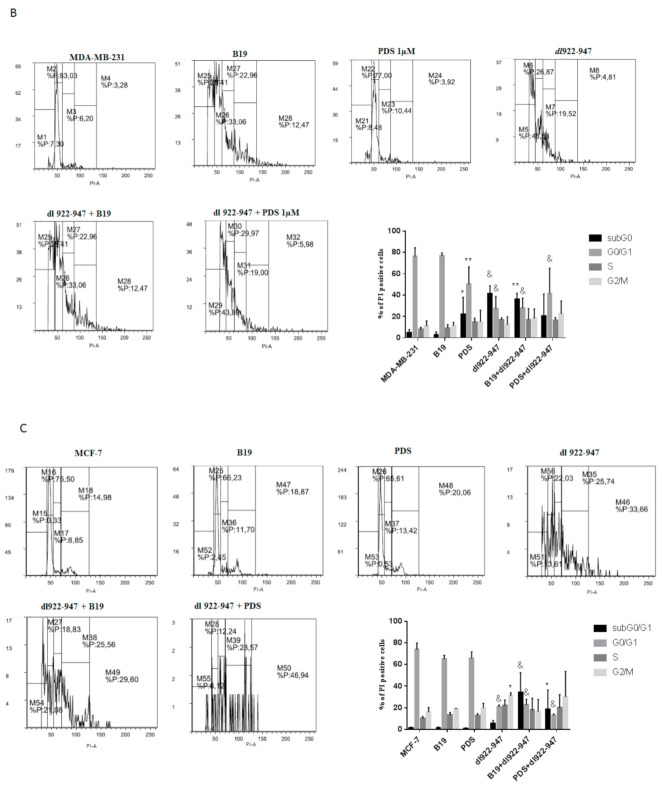
Cytotoxic effects of G4 ligands/*dl922-947* combination and regulation of the cell cycle progression. Cytotoxic effect of G4 ligands with *dl922-947* determined by SRB assays in MDA-MB-231 and MCF-7 cells after six days of treatment (**A**). Figures report cell death vs. untreated cells as mean of three independent experiments generated with IC_25_ and IC_50_ values of PDS and B19 (in µM) and IC_50_ value of *dl922-947* (in p.f.u.). The black bars represent the treatment with G4 binders alone, whereas the gray bars report the combination of *dl922-947* (at the IC_50_) with G4 ligands at the IC_25_ and/or IC_50_. Statistical significance was calculated by GraphPad Prism 7 with two-way ANOVA using Tukey’s multiple comparison test. * calculated versus untreated cells; $ calculated vs. PDS alone (IC_25_ and/or IC_50_); & calculated versus *dl922-947* alone; (*^&^ *p* < 0.05, ** *p* < 0.005, *** *p* < 0.0005, ****^,$^ *p* < 0.0001). MDA-MB-231 (**B**) and MCF-7 (**C**) cells were treated with *dl922-947*, G4 ligands or their combination at the IC_50_ values. After 6 days of treatment, cells were stained with PI. A representative flow cytometry profile of the cell cycle is shown for both cell lines, and the percent of cells in each phase of the cell cycle is indicated for the single experiment, whereas the bars in the histograms represent the mean ± SD of the least three independent experiments. The statistical significance was calculated by GraphPad Prism 7 with two-way ANOVA using Tukey’s multiple comparisons test (& *p* < 0.0001, ** *p* < 0.005, * *p* < 0.05).

We observed increase of cell death using *dl922-947*, B19, and PDS alone with respect to untreated cells (Figure 2A). In MDA-MB-231 cells, the combination *dl922-947* and PDS (IC_25_ and IC_50_), differently from the combination *dl922-947* and B19, increased cytotoxicity with respect to the single agents. This effect might support a correlation between higher cytotoxicity with higher G4 motif induction by PDS.

In MCF-7 cells, the combination of PDS or B19 with *dl922-947* (at the IC_50_ value) showed increased cell death; the effect was significant only with respect to the virus used alone (Figure 2A) and is consistent with G4 motif induction by both G4 ligands in this cell line.

### 3.4. dl922-947/G4 Binder Combination and Cell Cycle

The treatment with B19 at the IC_50_ value in both cell lines did not alter cell cycle profile with respect to the control, as well as PDS in MCF-7 cells. PDS at the IC_50_ induced cell accumulation in the subG0/G1 phase of the cell cycle in MDA-MB-231 cells (Figure 2B). The treatment with *dl922-947* decreased the G0/G1 phase and increased G2/M phase with respect to the control in MCF-7 cells (Figure 2C), while an increase of subG0/G1 phase and decrease of G1 phase was observed in MDA-MB-231 cells (Figure 2B). The combination *dl922-947*/G4 binders in MCF-7 cells increased the subG0/G1 and decreased G0/G1 phases with respect to the control (Figure 2C); in MDA-MB-231 cells, the combination produced an effect similar to that elicited by the virus alone, namely an increase of subG0/G1 phase and decrease of G1 phase (Figure 2B). PDS dose dependently decreased the G0/G1 phase of the cell cycle both in MDA-MB-231 and MCF-7 cells (Figure S3), indeed, in MCF-7 cells increased the subG0/G1 phase at the highest concentration. The combinatory treatments confirm an increased cell death as observed with cytotoxicity data.

### 3.5. G4-Binders Enhance Adenovirus Entry in Breast Cancer Cells

**Figure 3:**
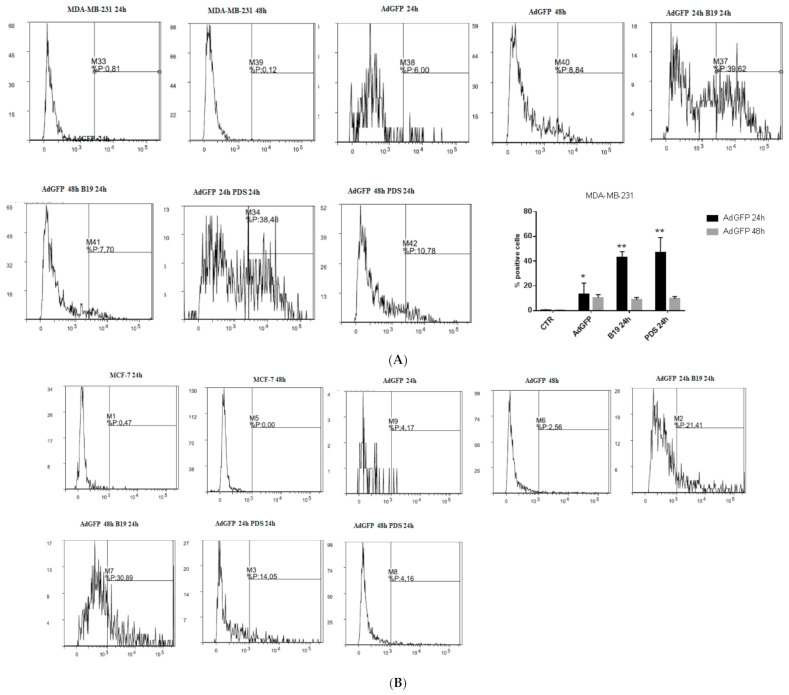

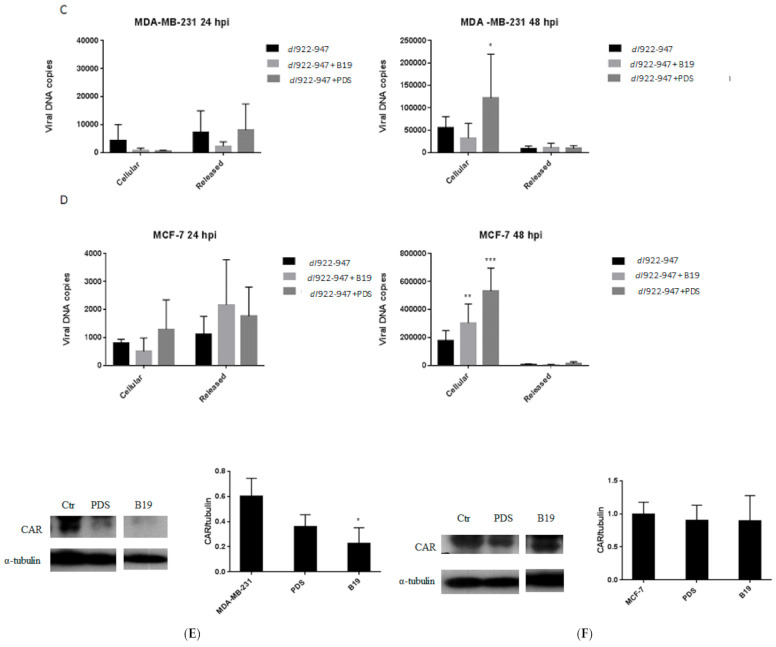
Adenovirus *Ad*GFP entry, *dl922-947* replication, and CAR modulation by G4 binders in breast cancer cells. MDA-MB-231 (**A**) and MCF-7 (**B**) cells were infected with the non-replicating *Ad*GFP and treated with G4 binders at different times. *Ad*GFP and G4 binders were added concomitantly (24 h after seeding the cells black bars **A** and **B**); or *Ad*GFP was added 24 h after G4 binders (*Ad*GFP was added 48 h after seeding cells, gray bars **A** and **B**). GFP emission was evaluated by flow cytometry and flow cytometry profile of a representative experiment is reported. The statistical significance was calculated by GraphPad Prism 7 with two-way ANOVA using Tukey’s multiple comparisons test (** *p* < 0.0001, * *p* < 0.05). The statistical significance is indicated in the histograms that represent the mean of at least three independent experiments. To evaluate viral replication, MDA-MB-231 (**C**) and MCF-7 cells (**D**) were treated with *dl922-947* (at the IC_50_ value) or *Ad*GFP (30 p.f.u. x cell, used as control at 48 hpi, not shown) in combination with G4 ligands (at their IC_50_ values). After 24 and 48 h of incubation, supernatants and adherent cells were collected separately to evaluate extracellular release and intracellular viral particles, respectively. Viral DNA was extracted and used to quantify viral titer by Real-Time qPCR. The data represent the mean of three independent experiments (**C**). Statistical significance was calculated comparing the increased intracellular amplification with respect to the correspondent extracellular fraction (* *p* < 0.05, ** *p* < 0.005, *** *p* < 0.0001). To evaluate CAR modulation by G4 binders, MDA-MB-231 (**E**) and MCF-7 cells (**F**) were treated with G4 binders (at the IC_50_ values) for 48 h. The images represent the expression of CAR evaluated by Western blot. As control, α-tubulin was used. Original blots are enclosed as supplemental Figure S5 for CAR. The histogram reports the mean of two independent experiments. The statistical significance was calculated by parametric paired two tailed *T* test, by GraphPad Prism 7 (* *p* < 0.05).

We evaluated whether G4 binders increased viral entry by analyzing the percent of cells expressing GFP after 48 h of incubation with the virus. In both MDA-MB-231 and MCF-7 cells, G4 binders added simultaneously with *Ad*GFP increased significantly viral entry (Figure 3A,B black bars). To evaluate the best experimental condition to increase viral entry, we incubated the cells with G4 binders and after a further 24 h, cells were infected with *Ad*GFP (30 p.f.u./cell). In MDA-MB-231 cells, the addition of *Ad*GFP after 24 h of incubation with G4 binders did not modify viral entry. In MCF-7 cells, an increase of viral entry was observed; however, the increase was statistically significant only with B19 (Figure 3A,B gray bars). A different manner of administration was performed adding G4 binders 24 h after *Ad*GFP and the increase of viral entry was maintained with both G4 ligands and in both the cell lines (Figure S4). In MCF-7 cells infected with *Ad*GFP alone, no significant increase of viral entry was observed (Figure 3B).

### 3.6. DNA Amplification of dl922-947

*dl922-947* amplification was evaluated in combination with B19 and/or PDS (at the IC_50_ values). After 24 hpi, the viral DNA was equally distributed in the intracellular and extracellular fractions. After 48 hpi, it was enhanced in the intracellular fraction, indicating an increase of intracellular viral DNA copies in the presence of G4 ligands (Figure 3C,D). In particular, the intracellular amplification was observed using *dl922-947* in combination with PDS but not with B19 in MDA-MB-231 cells (Figure 3C) and with both G4 ligands in MCF-7 cells (Figure 3D). No DNA amplification after 48 h of treatment was detected using the non-replicating *Ad*GFP as negative control (data not shown).

### 3.7. G4 Binder Effect on Coxsackie-Adenovirus Receptor (CAR) Expression

We evaluated the potential modulation by G4 binders of CAR expression, a known site of adenovirus attachment during infection that also showed elevated expression in breast cancer cells [30]. After 48 h of treatment, we observed in MDA-MB-231 cells slight decrease of CAR expression by G4 binders at the IC_50_ that reached statistical significance with B19 (Figure 3E). In MCF-7 cells, G4 binders did not modify CAR expression (Figure 3F).

### 3.8. dl922-947 and Its Combination with PDS Induce G4 Motifs in MCF-7 Cells

**Figure 4:**
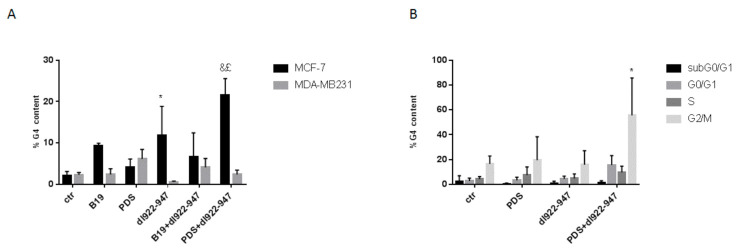
Induction of G4 motif content and distribution in cell cycle phases. G4 motif induction was evaluated by flow cytometry. MCF-7 and MDA-MB-231 cells were treated with *dl922-947*, G4 binders, or their combination at the IC_50_ values for 6 days. The histograms represent the mean ± SD of at least three independent experiments (**A**). Gating strategies were adopted to select in each phase of the cell cycle the percent of G4 structure positivity in MCF-7 cells (**B**). The statistical significance was calculated by GraphPad Prism 7 with two-way ANOVA using Sidak’s multiple comparisons test (* *p* < 0.001, & *p* < 0.0001; £ *p* < 0.05; * = statistical significance calculated vs. ctr, & = statistical significance calculated vs. PDS, £ = statistical significance calculated vs. *dl922-947*).

We investigated potential modifications in G4 motif formation using the combination *dl922-947* and G4 binders. Surprisingly, we observed that *dl922-947* alone favored G4 motif formation in MCF-7 cells and the effect was potentiated by PDS (Figure 4A), but not by B19. G4 motif formation was observed in G2/M phase of the cell cycle (reported for the combination *dl922-947* and PDS in MCF-7 cells) with respect to the control (untreated cells) and to the adenovirus and PDS used separately (Figure 4B). No induction of G4 motifs was observed in MDA-MB-231 cells with the combination G4 binders and virus (Figure 4A).

### 3.9. Induction of Senescence

**Figure 5:**
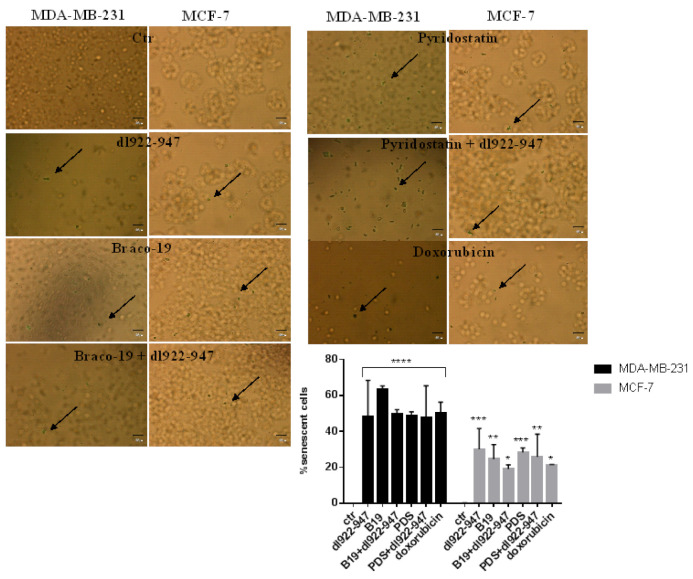
Induction of cell senescence. MDA-MB-231 and MCF-7 cells were treated for 72 h with *dl922-947*, G4 binders, or their combination at the IC_50_ values. Senescent cells are indicated in the images by the arrows as blue SA-b-Gal positive stained cells. Doxorubicin was used as positive control. The images are representative of three independent experiments. The statistical significance indicated in the histograms was calculated by GraphPad Prism 7 with two-way ANOVA with Tukey’s multiple comparisons test (**** *p* < 0.0001, *** *p* < 0.001, ** *p* < 0.01, * *p* < 0.05).

G4 binders are well known inducers of cell senescence, therefore we analyzed the senescence induction in the presence of *dl922-947*. Interestingly, we observed that *dl922-947* induced cell senescence, however, its combination with G4 binders did not increase the effect. Notably, the induction of senescence was higher in MDA-MB-231 than in MCF-7 cells (Figure 5). The effects observed were compared to doxorubicin known to induce senescence in breast cancer cells.

### 3.10. STING Induction by G4 Binders in Breast Cancer Cells

**Figure 6:**
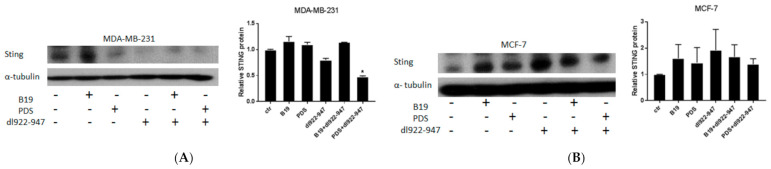
Expression of STING in breast cancer cells. The image represents the expression of STING evaluated by Western blot in MDA-MB-231 cells (**A**) and MCF-7 cells (**B**) treated for 24 h with G4 binders, *dl922-947*, and their combination at the IC_50_. As control, α-tubulin was used. The histogram reports the mean of two or three independent experiments, respectively, for MDA-MB-231 and MCF-7 cells. Original blots are enclosed as supplemental Figure S6 for STING. The statistical significance was calculated by parametric paired two tailed *t* test, by GraphPad Prism 7 (* *p* < 0.05).

Furthermore, we investigated the modulation of stimulator of interferon genes (STING) pathway known to promote pro-tumor and anti-tumor activity depending on the tumor context [31]. In MDA-MB-231 cells, we detected a slight decreased expression of STING using *dl922-947*, although the result did not reach statistical significance, while the combination *dl922-947*/PDS in a statistically significant manner reduced STING expression compared to the untreated control cells, the virus, and PDS alone (Figure 6A). In MCF-7 cells, we observed a weak increase of the expression of STING in MCF-7 cells by G4 binders, *dl922-947*, and their combination although the effect did not reach the statistical significance (Figure 6B).

## 4. Discussion

Although the role of G4 motifs in living cells was controversially discussed, accumulating evidence supports the relevance of these structures [32]. Studies in the last years have suggested that the stabilization of G4 structure by G4 ligands might be useful as novel therapeutic tool in cancer treatment [33]. In the present study, we evaluated the anticancer efficacy in breast cancer cells of B19 and PDS alone and in combination with the oncolytic adenovirus *dl922-947*. Differently from B19, PDS is able to target, among others, the G4 motifs in the *SRC* proto-oncogene [21]. We have previously observed the inhibitory effect of B19 in MDA-MB-231 and MCF-7 cell proliferation [7]. In a panel of neoplastic cell lines, we observed that B19 and PDS were particularly efficient at inhibiting cell survival in MDA-MB-231 and MCF-7 cells.

We observed that B19 and PDS elicited cytotoxicity in MDA-MB-231 cells at similar IC_50_ doses. The IC_50_ doses of both drugs were generally higher in MCF-7 cells compared to MDA-MB-231, and the IC_50_ values of PDS were lower with respect to B19. These data indicate that MDA-MB-231 cells might be more sensitive to G4 binder treatments. It might be speculated that highly proliferative and aggressive cell lines, given their high number of cell division, might more and more rapidly form G4 motifs, explaining the lower concentrations required with respect to MCF-7 cells.

We evaluated potential differences in G4 motif stabilization by the two drugs. In MDA-MB-231 cells, PDS was more efficient with respect to B19 in stabilizing G4 motifs, while in MCF-7 cells with both drugs similar G4 motif levels were observed. The different levels of G4 motifs observed in the presence of B19 and PDS in MDA-MB-231 cells suggest that these two G4 binders might differently interact with G4 structures and that not all the interactions are effective in stabilizing G4 motifs. This hypothesis might be supported by our recent study suggesting that the binding mechanism of B19 and PDS to G4 motifs is very different; we observed that the binding of PDS to G4 motifs occurred mainly via loop interactions, while for B19 it depended on G4 motif conformations [6]. The evaluation of G4 motif distribution in the different cell cycle phases showed in MDA-MB-231 cells a distribution of G4 motifs in S and G2/M phases with a statistically significant increase compared to the control only for PDS in the S phase.

In MCF-7 cells, we observed an increase of S and G2/M phases for both the agents and a significant increase also in the subG0/G1 phase by B19. This observation is not in agreement with a previous study showing G4 motifs in MDA-MB-231 cells treated with B19 only in subG0/G1 phase, however, in that study, higher concentrations of B19 and a less specific G4-detecting antibody were used [7]. However, the presence of G4 motifs in MCF-7 cells in the subG0/G1 phase suggests that apoptotic cells present G4 motifs, since we previously described apoptosis induction by B19 in this cell line [7].

G4 ligands induce genome instability and subsequent DNA damage [7] that might favor or potentiate the anticancer activity of other agents such as the oncolytic adenovirus *dl922-947*. We demonstrated in the malignant mesothelioma that *dl922-947* synergized with cisplatin [11], which is known to target duplex DNA but also to react with G4 motifs [34]. We addressed potential increase of cytotoxicity using the combination *dl922-947/*G4 ligands. We observed in MDA-MB-231 cells that PDS in combination with the adenovirus enhanced cytotoxicity with respect to both agents used alone. Further studies will be addressed to investigating additive/synergic effects. No significant difference using the combination *dl922-947*/B19 with respect to the agents used alone was observed. In MCF-7 cells, the combination of both G4 ligands with *dl922-947* enhanced cytotoxicity with respect to the virus used alone. These results are in agreement with the increase of the subG0/G1 phase of the cell cycle using virus/G4 binder combinations in both MCF-7 and MDA-MB-231 cells. The increase in subG0/G1 phases correlated with a decrease of the G1 phase that suggests a reduced cycle progression with accumulation in subG0/G1 phase. Additionally, our results suggest that higher cytotoxicity as observed using PDS/*dl922-947* combination correlated with higher stabilization of G4 motifs in both cell lines. Conversely, B19/*dl922-947* combination did not enhance cytotoxicity and G4 motif formation in MDA-MB-231 cells, whereas in MCF-7 cells, the enhanced cytotoxicity was accompanied by formation of G4 motifs. Moreover, we investigated if potentiated effects in the combinatory treatments were associated to increased viral entrance and replication. We observed that the virus is able to enter MDA-MB-231 cells more efficiently than in MCF-7 cells. However, the simultaneous co-administration with G4 ligands increased viral entry also in MCF-7 cells. Notably, we observed in MDA-MB-231 cells increased DNA amplification in the intracellular fraction with respect to the extracellular fraction using the combination PDS/*dl922-947*. In MCF-7 cells, the combination with both G4 ligands enhanced viral copies in the intracellular fraction. These results further support and correlate with the effects observed with G4 motif formation. In both cell lines in which PDS enhanced G4 motifs, we observed increased viral replication, while B19 stabilized G4 motifs only in MCF-7 cells and only in this cell line, it supported viral replication. Overall, these data suggest that G4 motif formation might favor viral replication. In order to address a potential mechanism involved in the increase of viral entry and replication by G4 binders, we analyzed CAR modulation by G4 binders. CAR was the first adenovirus receptor identified allowing adenovirus cellular entry in co-operation with cell surface integrin receptors; however, alternative adenovirus receptors have been described such as CD46, desmoglein-2 receptors (DSG-2), and sialic acid [35]. In MDA-MB-231 cells, the decrease of CAR observed with B19 is in agreement with the low and comparable replicative potential induced by B19 with *dl922-947* alone. No significant effect was elicited by PDS in CAR modulation in MDA-MB-231 cells. In MCF-7 cells, no modulation of CAR expression was observed by G4 binders. These results suggest that alternative receptors as above mentioned might be also involved. Indeed, it was also reported that CAR expression increases during breast cancer progression and metastasis and enhances breast cancer cell proliferation [36]. The absence of upregulation of CAR expression might be consistent with the anticancer activity of G4 binders.

Furthermore, we investigated the stabilization of G4 motifs using *dl922-947*/G4 binder combination. Surprisingly, *dl922-947* was able to stabilize G4 motif t in MCF-7 cells, and this effect was enhanced in the presence of PDS. G4 motif distribution upon *dl922-947* or *dl922-947*/PDS combination was detected in the G2/M phase of the cell cycle.

In MDA-MB-231 cells, the virus did not stabilize G4 motifs likely because highly proliferating cells might more and more rapidly form G4 motifs difficult to stabilize. It might be speculated that the absence of G4 motifs might be in contrast with the cytotoxic effect observed in MDA-MB-231 cells with the combination virus/PDS as we demonstrated that PDS stabilizes G4 motifs and is able to provide a better viral entrance and replication. However, the cytotoxic effect observed might also be due to the induction of cell death mechanisms induced by the single agents beyond G4 motifs.

Since G4 stabilization can be accompanied by cell senescence [37], we investigated if *dl922-947* or its combination with G4 binders induced/potentiated cell senescence. Surprisingly, *dl922-947* alone induced cell senescence in both cell lines and its effect was similar to that elicited by G4 binders. The combination virus/G4 binders did not modify the senescence profile. Our data represent the first evidence that the adenovirus *dl922-947* induces G4 motifs and senescence in cancer cells. The induction of senescence by *dl922-947* is independent on G4 motif stabilization.

Recent findings, suggested that the accumulation of cytoplasmatic DNA in senescent cells activates the cGAS–STING pathway. Indeed, the dual role in cancer of this pathway is known since it can promote or inhibit tumorigeneses according to the biological context [38]. A previous study showed that STING pathway was induced by PDS in MCF-7 cells [39]. In agreement with this study, we observed that G4 ligands slightly increased STING expression and the effect was maintained with the virus and the combinatory treatments (although without reaching statistical significance). These data suggest that in MCF-7 cells, the activation of STING pathway, known to be related to apoptotic signaling in breast cancer cells [31], might be involved in the induction of apoptosis that we previously observed in MCF-7 cells treated with B19 [7]. In MCF-7 cells, the weak activation of STING might play an anti-tumor role. In contrast, in MDA-MB-231 cells, we observed that the *dl922-947* alone (although with no statistically significant results) and the combination *dl922-947*/PDS reduced STING signaling. A very recent study showed that STING signaling inactivation impaired the survival of triple negative breast cancer cells. The chromosomal instability in these cells leads to DNA release by micronuclei into the cytoplasm promoting STING activation and cancer progression [40]. In this context, our data suggest that the combination *dl922-947*/PDS might promote an anti-tumor role by inactivating STING signaling independently on G4 structures. Further investigations are required to understand these effects by the analyses of multiple pathways potentially involved in STING signaling and mediators regulating its activation.

In conclusion, our results suggest different mechanisms of action elicited by the adenovirus alone and in combination with G4 binders in different breast cancer cells. Further investigations are required to understand these effects. However, we provided evidence that G4 binders might be good candidates to use as single therapy against breast cancer, or with virotherapy in types of tumors suitable or insensitive to OVs.

## 5. Conclusions

These data propose a never-investigated anticancer approach that combines virotherapy and G-quadruplex (G4) motifs. Both anticancer therapies separately applied, virotherapy based on the use of oncolytic viruses (OVs), and the use of G4-targeting molecules revealed interest in a clinical setting. In breast cancer cells, the combination of OVs and G4 binders enhances the cytotoxic effects, viral entry, and replication. G4 ligands and the adenovirus *dl922-947* induce breast cancer cell senescence and surprisingly *dl922-947* stabilizes G4 motifs, an effect potentiated by PDS. Indeed, the combination *dl922-947/*PDS inactivates STING signaling pathways consistently with antitumor activity. These data provide new knowledge about the mechanism of action of the *dl922-947* and propose that its combination with G4 ligands might promote the use of virotherapy also in types of tumor not sensitive to OV treatment. Our approach based on already known antitumor agents highlights that this novel combinatory strategy might likely find a translation to the clinical setting.

## Figures and Tables

**Table 1:** (A) Comparison of IC_50_ values on different cancer cell lines treated with B19. Cytotoxic effect of B19 by SRB assays after 6 days of treatment was tested in different cancer cell lines (MDA-MB-231, MCF-7, BHT, and PC3) using increasing concentrations. The table reports the IC_50_ values calculated by GraphPad Prism 7. (B) Comparison of IC_25_ and IC_50_ values in breast cancer cell lines treated with B19, PDS, and *dl922-947*. Table reports IC_25_ and IC_50_ values calculated form dose-response curves of B19, PDS, and *dl922-947* using increasing doses of the agents in MDA-MB-231 and MCF-7 cells after 6 days of treatment. For G4 ligands, the doses are expressed in µM, for *dl922-947* in p.f.u.

A			
MDA-MB-231		1.1 µM	
MCF-7		2.5 µM	
BHT-101		3.1 µM	
PC3		4.6 µM	
B			
	**MDA-MB-231**		**MCF-7**
B19 IC25	0.3 µM		1.2 µM
B19 IC50	1.1 µM		2.5 µM
PDS IC25	0.5 µM		0.8 µM
PDS IC50	1.0 µM		1.5 µM
*dl922-947* IC25	0.5 p.f.u.		0.1 p.f.u.
*dl922-947* IC50	0.8 p.f.u.		0.3 p.f.u.

## Data Availability

The data supporting the findings of this study are available from the corresponding author upon reasonable request.

## Supplementary Materials

The following supporting information can be downloaded at: https://www.mdpi.com/article/10.3390/cells11162482/s1.

## References

[1] Amato J., Miglietta G., Morigi R., Iaccarino N., Locatelli A., Leoni A., Novellino E., Pagano B., Capranico G., Randazzo A. (2020). Monohydrazone Based G-Quadruplex Selective Ligands Induce DNA Damage and Genome Instability in Human Cancer Cells. J. Med. Chem..

[2] Amato J., Pagano A., Capasso D., Di Gaetano S., Giustiniano M., Novellino E., Randazzo A., Pagano B. (2018). Targeting the BCL2 Gene Promoter G-Quadruplex with a New Class of Furopyridazinone-Based Molecules. ChemMedChem.

[3] Burge S., Parkinson G.N., Hazel P., Todd A.K., Neidle S. (2006). Quadruplex DNA: Sequence, topology and structure. Nucleic Acids Res..

[4] Hänsel-Hertsch R., Di Antonio M., Balasubramanian S. (2017). DNA G-quadruplexes in the human genome: Detection, functions and therapeutic potential. Nat. Rev. Mol. Cell Biol..

[5] Nakanishi C., Seimiya H. (2020). G-quadruplex in cancer biology and drug discovery. Biochem. Biophys. Res. Commun..

[6] Ivancich M., Schrank Z., Wojdyla L., Leviskas B., Kuckovic A., Sanjali A., Puri N. (2017). Treating Cancer by Targeting Telomeres and Telomerase. Antioxidants.

[7] Di Somma S., Amato J., Iaccarino N., Pagano B., Randazzo A., Portella G., Malfitano A.M. (2019). G-Quadruplex Binders Induce Immunogenic Cell Death Markers in Aggressive Breast Cancer Cells. Cancers.

[8] Malfitano A.M., Di Somma S., Iannuzzi C.A., Pentimalli F., Portella G. (2020). Virotherapy: From single agents to combinatorial treatments. Biochem. Pharmacol..

[9] Malfitano A.M., Pisanti S., Napolitano F., Di Somma S., Martinelli R., Portella G. (2020). Tumor-Associated Macrophage Status in Cancer Treatment. Cancers.

[10] Di Somma S., Napolitano F., Portella G., Malfitano A.M. (2021). Cross Talk of Macrophages with Tumor Microenvironment Cells and Modulation of Macrophages in Cancer by Virotherapy. Biomedicines.

[11] Di Somma S., Iannuzzi C.A., Passaro C., Forte I.M., Iannone R., Gigantino V., Indovina P., Botti G., Giordano A., Formisano P. (2019). The Oncolytic Virus *dl922-947* Triggers Immunogenic Cell Death in Mesothelioma and Reduces Xenograft Growth. Front. Oncol..

[12] Passaro C., Borriello F., Vastolo V., Di Somma S., Scamardella E., Gigantino V., Franco R., Marone G., Portella G. (2016). The oncolytic virus *dl922-947* reduces IL-8/CXCL8 and MCP-1/CCL2 expression and impairs angiogenesis and macrophage infiltration in anaplastic thyroid carcinoma. Oncotarget.

[13] Bazan-Peregrino M., Carlisle R.C., Hernandez-Alcoceba R., Iggo R., Homicsko K., Fisher K.D., Halldén G., Mautner V., Shen Y., Seymour L.W. (2008). Comparison of molecular strategies for breast cancer virotherapy using oncolytic adenovirus. Hum. Gene Ther..

[14] Du W., Searle J.S. (2009). The rb pathway and cancer therapeutics. Curr. Drug Targets.

[15] Libertini S., Abagnale A., Passaro C., Botta G., Barbato S., Chieffi P., Portella G. (2011). AZD1152 negatively affects the growth of anaplastic thyroid carcinoma cells and enhances the effects of oncolytic virus *dl922-947*. Endocr.-Relat. Cancer.

[16] Iannuzzi C.A., Indovina P., Forte I.M., Di Somma S., Malfitano A.M., Bruno M., Portella G., Pentimalli F., Giordano A. (2020). Pharmacological Inhibition of WEE1 Potentiates the Antitumoral Effect of the *dl922-947* Oncolytic Virus in Malignant Mesothelioma Cell Lines. Int. J. Mol. Sci..

[17] Passaro C., Volpe M., Botta G., Scamardella E., Perruolo G., Gillespie D., Libertini S., Portella G. (2015). PARP inhibitor olaparib increases the oncolytic activity of *dl922-947* in in vitro and in vivo model of anaplastic thyroid carcinoma. Mol. Oncol..

[18] Botta G., Passaro C., Libertini S., Abagnale A., Barbato S., Maione A.S., Hallden G., Beguinot F., Formisano P., Portella G. (2012). Inhibition of autophagy enhances the effects of E1A-defective oncolytic adenovirus *dl922-947* against glioma cells in vitro and in vivo. Hum. Gene Ther..

[19] Libertini S., Iacuzzo I., Perruolo G., Scala S., Ieranò C., Franco R., Hallden G., Portella G. (2008). Bevacizumab increases viral distribution in human anaplastic thyroid carcinoma xenografts and enhances the effects of E1A-defective adenovirus *dl922-947*. Clin. Cancer Res..

[20] Passaro C., Abagnale A., Libertini S., Volpe M., Botta G., Cella L., Pacelli R., Halldèn G., Gillespie D., Portella G. (2013). Ionizing radiation enhances *dl922-947*-mediated cell death of anaplastic thyroid carcinoma cells. Endocr.-Relat. Cancer.

[21] Rodriguez R., Miller K.M., Forment J.V., Bradshaw C.R., Nikan M., Britton S., Oelschlaegel T., Xhemalce B., Balasubramanian S., Jackson S.P. (2012). Small-molecule-induced DNA damage identifies alternative DNA structures in human genes. Nat. Chem. Biol..

[22] Botta G., Perruolo G., Libertini S., Cassese A., Abagnale A., Beguinot F., Formisano P., Portella G. (2010). PED/PEA-15 modulates coxsackievirus-adenovirus receptor expression and adenoviral infectivity via ERK-mediated signals in glioma cells. Hum. Gene Ther..

[23] Malfitano A.M., Sosa S., Laezza C., De Bortoli M., Tubaro A., Bifulco M. (2011). Rimonabant reduces keratinocyte viability by induction of apoptosis and exerts topical anti-inflammatory activity in mice. Br. J. Pharmacol..

[24] Manna S., Florio D., Iacobucci I., Napolitano F., Benedictis I., Malfitano A.M., Monti M., Ravera M., Gabano E., Marasco D. (2021). A Comparative Study of the Effects of Platinum (II) Complexes on β-Amyloid Aggregation: Potential Neurodrug Applications. Int. J. Mol. Sci..

[25] Laezza C., Malfitano A.M., Di Matola T., Ricchi P., Bifulco M. (2010). Involvement of Akt/NF-κB pathway in N6-isopentenyladenosine-induced apoptosis in human breast cancer cells. Mol. Carcinog..

[26] Malfitano A.M., Laezza C., Pisanti S., Gazzerro P., Bifulco M. (2008). Rimonabant (SR141716) exerts anti-proliferative and immunomodulatory effects in human peripheral blood mononuclear cells. Br. J. Pharmacol..

[27] Pisanti S., Picardi P., Ciaglia E., Margarucci L., Ronca R., Giacomini A., Malfitano A.M., Casapullo A., Laezza C., Gazzerro P. (2014). Antiangiogenic effects of N6-isopentenyladenosine, an endogenous isoprenoid end product, mediated by AMPK activation. FASEB J..

[28] Ciaglia E., Pisanti S., Picardi P., Laezza C., Malfitano A.M., D’Alessandro A., Gazzerro P., Vitale M., Carbone E., Bifulco M. (2013). N6-isopentenyladenosine, an endogenous isoprenoid end product, directly affects cytotoxic and regulatory functions of human NK cells through FDPS modulation. J. Leukoc. Biol..

[29] De Magis A., Kastl M., Brossart P., Heine A., Paeschke K. (2021). BG-flow, a new flow cytometry tool for G-quadruplex quantification in fixed cells. BMC Biol..

[30] Martin T.A., Watkins G., Jiang W.G. (2005). The Coxsackie-adenovirus receptor has elevated expression in human breast cancer. Clin. Exp. Med..

[31] Lohard S., Bourgeois N., Maillet L., Gautier F., Fétiveau A., Lasla H., Nguyen F., Vuillier C., Dumont A., Moreau-Aubry A. (2020). STING-dependent paracriny shapes apoptotic priming of breast tumors in response to anti-mitotic treatment. Nat. Commun..

[32] Varshney D., Spiegel J., Zyner K., Tannahill D., Balasubramanian S. (2020). The regulation and functions of DNA and RNA G-quadruplexes. Nat. Rev. Mol. Cell Biol..

[33] Carvalho J., Mergny J.L., Salgado G.F., Queiroz J.A., Cruz C. (2020). G-quadruplex, Friend or Foe: The Role of the G-quartet in Anticancer Strategies. Trends Mol. Med..

[34] Ju H.P., Wang Y.Z., You J., Hou X.M., Xi X.G., Dou S.X., Li W., Wang P.Y. (2016). Folding Kinetics of Single Human Telomeric G-Quadruplex Affected by Cisplatin. ACS Omega.

[35] Mach N., Gao J., Schaffarczyk L., Janz S., Ehrke-Schulz E., Dittmar T., Ehrhardt A., Zhang W. (2020). Spectrum-Wide Exploration of Human Adenoviruses for Breast Cancer Therapy. Cancers.

[36] Vindrieux D., Le Corre L., Hsieh J.T., Métivier R., Escobar P., Caicedo A., Brigitte M., Lazennec G. (2011). Coxsackie and adenovirus receptor is a target and a mediator of estrogen action in breast cancer. Endocr.-Relat. Cancer.

[37] Long W., Zheng B.X., Li Y., Huang X.H., Lin D.M., Chen C.C., Hou J.Q., Ou T.M., Wong W.L., Zhang K. (2022). Rational design of small-molecules to recognize G-quadruplexes of c-MYC promoter and telomere and the evaluation of their in vivo antitumor activity against breast cancer. Nucleic Acids Res..

[38] Loo T.M., Miyata K., Tanaka Y., Takahashi A. (2020). Cellular senescence and senescence-associated secretory phenotype via the cGAS-STING signaling pathway in cancer. Cancer Sci..

[39] Miglietta G., Russo M., Duardo R.C., Capranico G. (2021). G-quadruplex binders as cytostatic modulators of innate immune genes in cancer cells. Nucleic Acids Res..

[40] Hong C., Schubert M., Tijhuis A.E., Requesens M., Roorda M., van den Brink A., Ruiz L.A., Bakker P.L., van der Sluis T., Pieters W. (2022). cGAS-STING drives the IL-6-dependent survival of chromosomally instable cancers. Nature.

