# Supplementary material for: Combination of dl922-947 Oncolytic Adenovirus and G-Quadruplex Binders Uncovers Improved Antitumor Activity in Breast Cancer
Source: Cells. 2022 Aug 10;11(16):2482. doi: 10.3390/cells11162482 (PMC9406944; doi:10.3390/cells11162482)
Supplement: Supplementary file 1 [file cells-11-02482-s001.zip › cells-1785678-supplementary.pdf]

SUPPLEMENTARY FIGURES

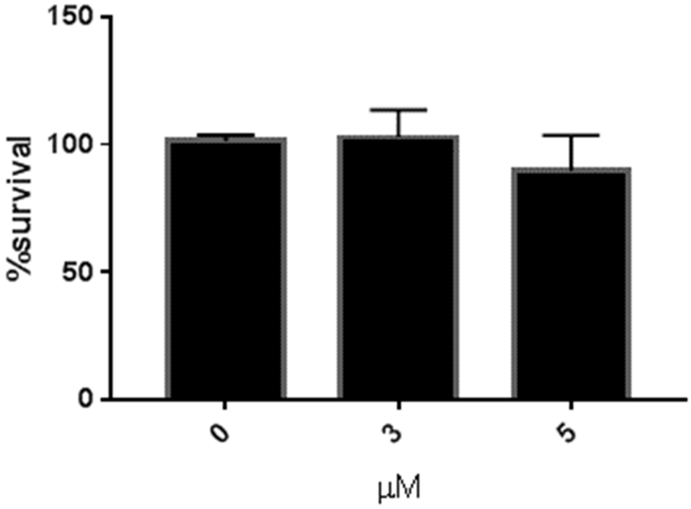

Figure S1. B19 treatment in MCF-10A cells.

Effect of B19 determined by SRB assays in MCF-10A cell survival after 6 days of treatment. Figure reports cell viability (% of survival) at the concentrations of B19 indicated. No statistical difference (calculated by GraphPad Prism 7 with two-way Anova using Sidak's multiple comparisons) was observed with respect to the untreated control.

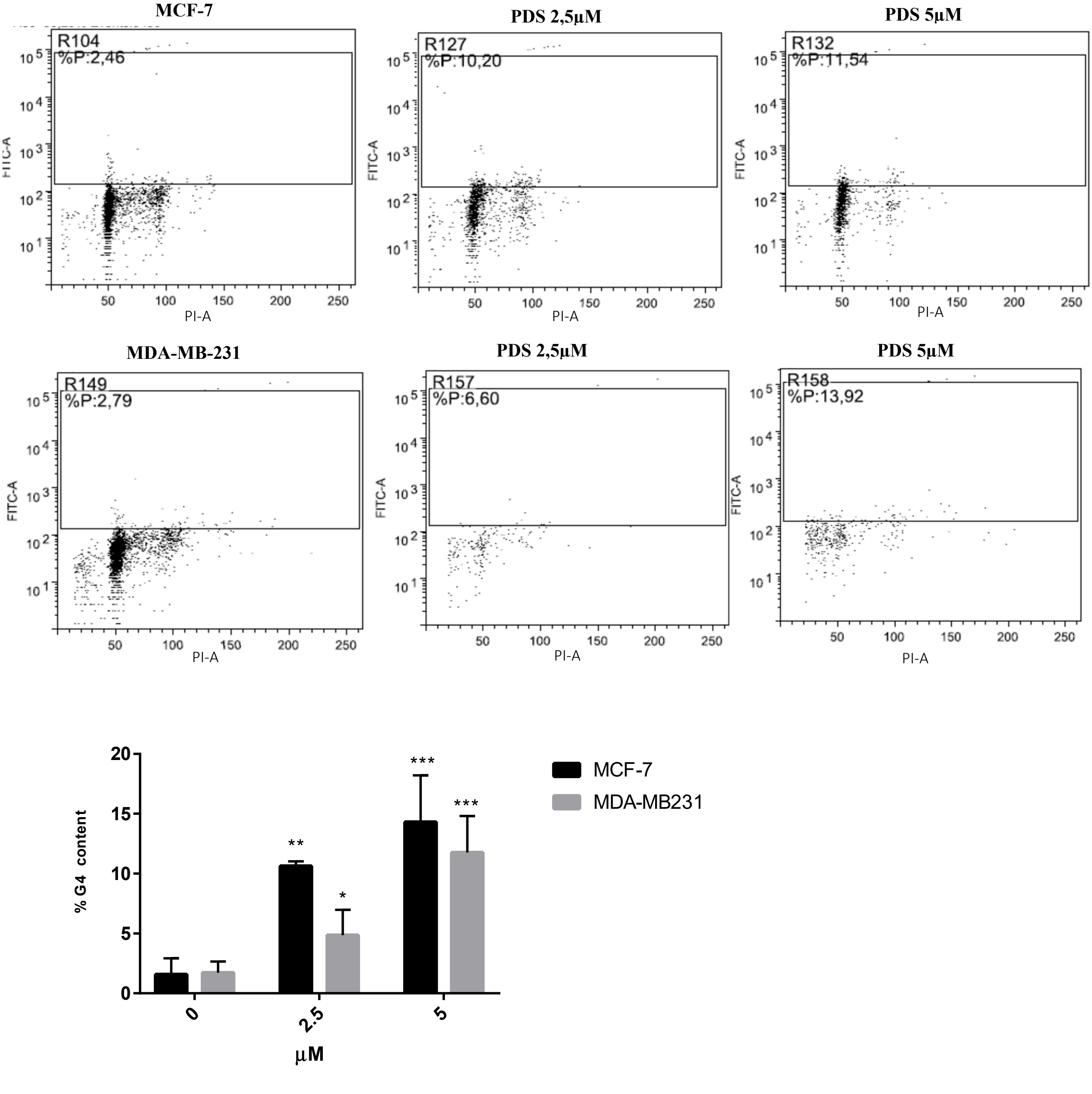

Figure S2.

G4 motif induction by PDS.

MCF-7 and MDA-MB-231 cells were treated with G4 ligands at the indicated concentrations. The dot plot profiles indicate in the upper FITC positive quadrant the content of G4 structures on the DNA of (PI positive) the cells. The dot plots reported are representative of a single experiment, whereas the histograms represent the mean ±SD of at least three independent experiments. The statistical significance was calculated by GraphPad Prism 7 with two-way ANOVA using Dunnet's multiple comparisons test (***p<0.0001, **p< 0.0005, *p<0.05).

A

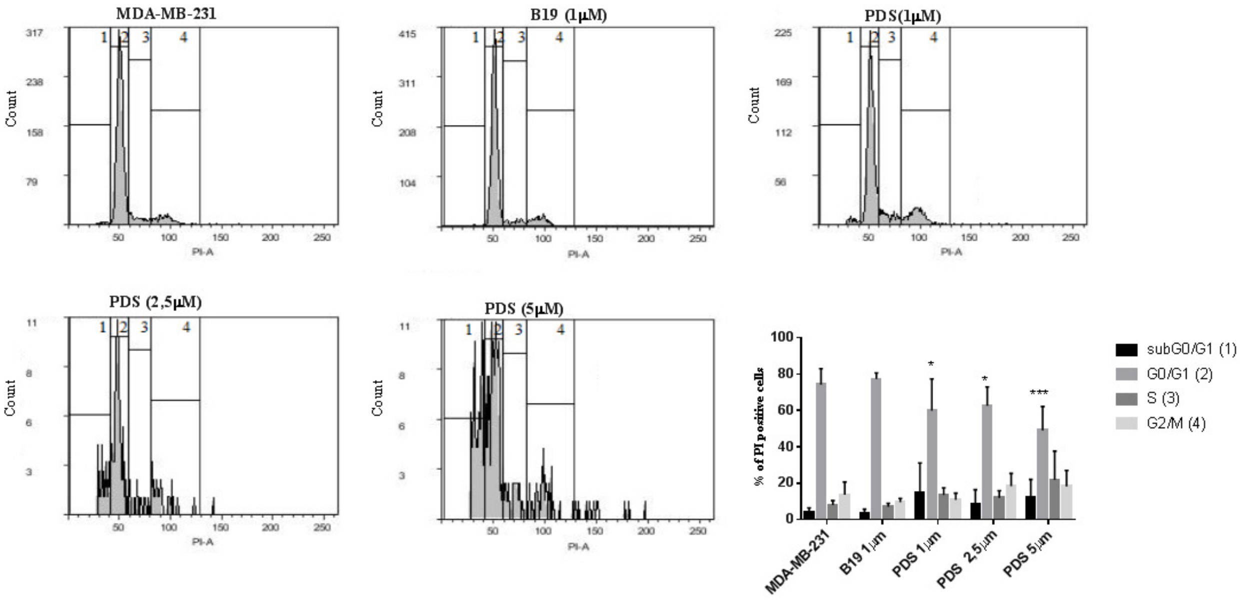

B

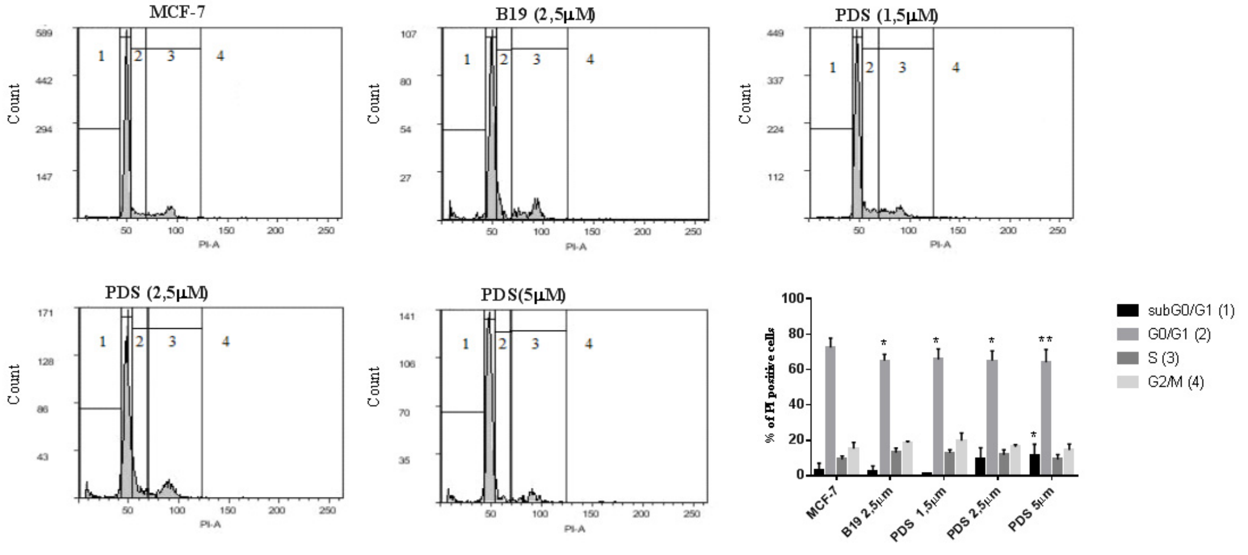

Figure S3. Regulation of cell cycle progression by G4 ligands.

MDA-MB-231 (A) and MCF-7 (B) cells were treated with G4 ligands at the indicated concentrations for 6 days. Cells were stained with PI to evaluate cell cycle progression. A representative flow cytometry profile of cell cycle is shown for both cell lines and the percent of cells in each phase of the cell cycle is indicated for a single experiment, whereas the bars in the histograms represent the mean ±SD of at least three independent experiments. The statistical significance was calculated by GraphPad Prism 7 with two-way ANOVA using Dunnet's multiple comparisons test (***p ≤0.0001,** p < 0.01, *p< 0,05).

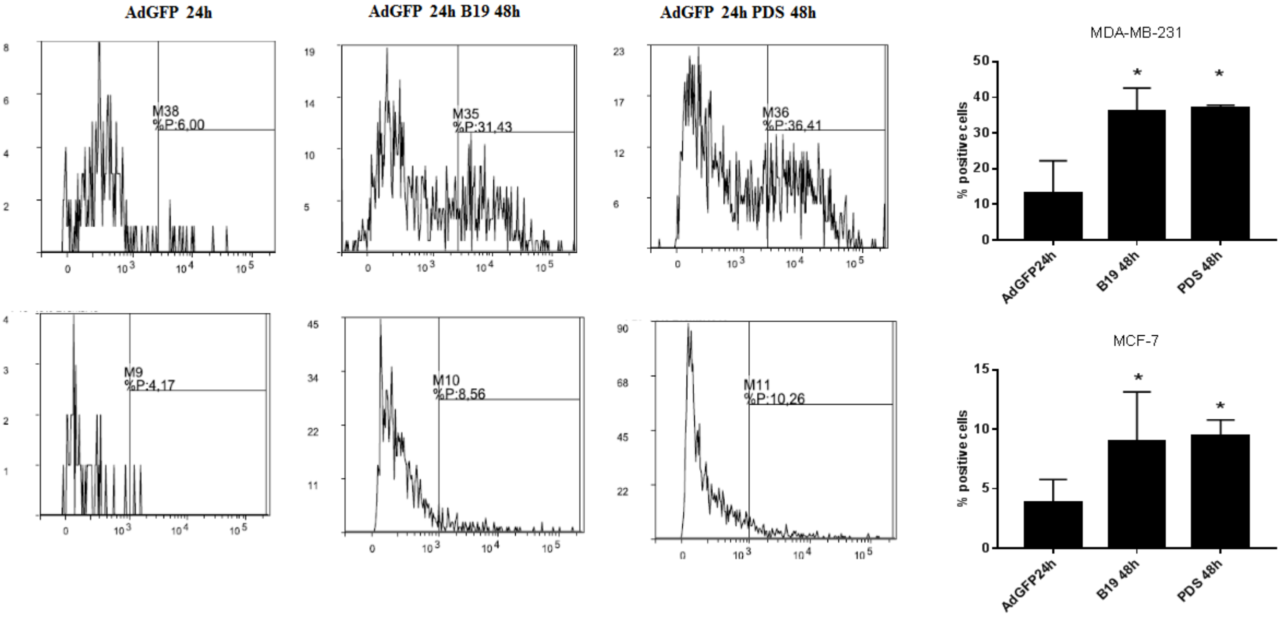

Figure S4. G4 binders increase adenovirus AdGFP entry.

G4 binders were added 24 h after AdGFP (AdGFP was added 24 h after seeding the cells); the histograms report the control (AdGFP 24h) for both MDA-MB-231 and MCF-7 cells and the treatment with B19 and PDS added 48h after seeding the cells and 24h after the addition of AdGFP (AdGFP 24h B19 48h, AdGFP 24h PDS 48h). GFP emission was evaluated by flow cytometry. The statistical significance was calculated by GraphPad Prism 7 with two-way ANOVA using Tukey's multiple comparisons test (**$p < 0.0001$, *$p < 0.05$) The statistical significance is indicated in the bar histograms that represent the mean of at least three independent experiments and are placed next to the flow cytometric profile representative of a single experiment.

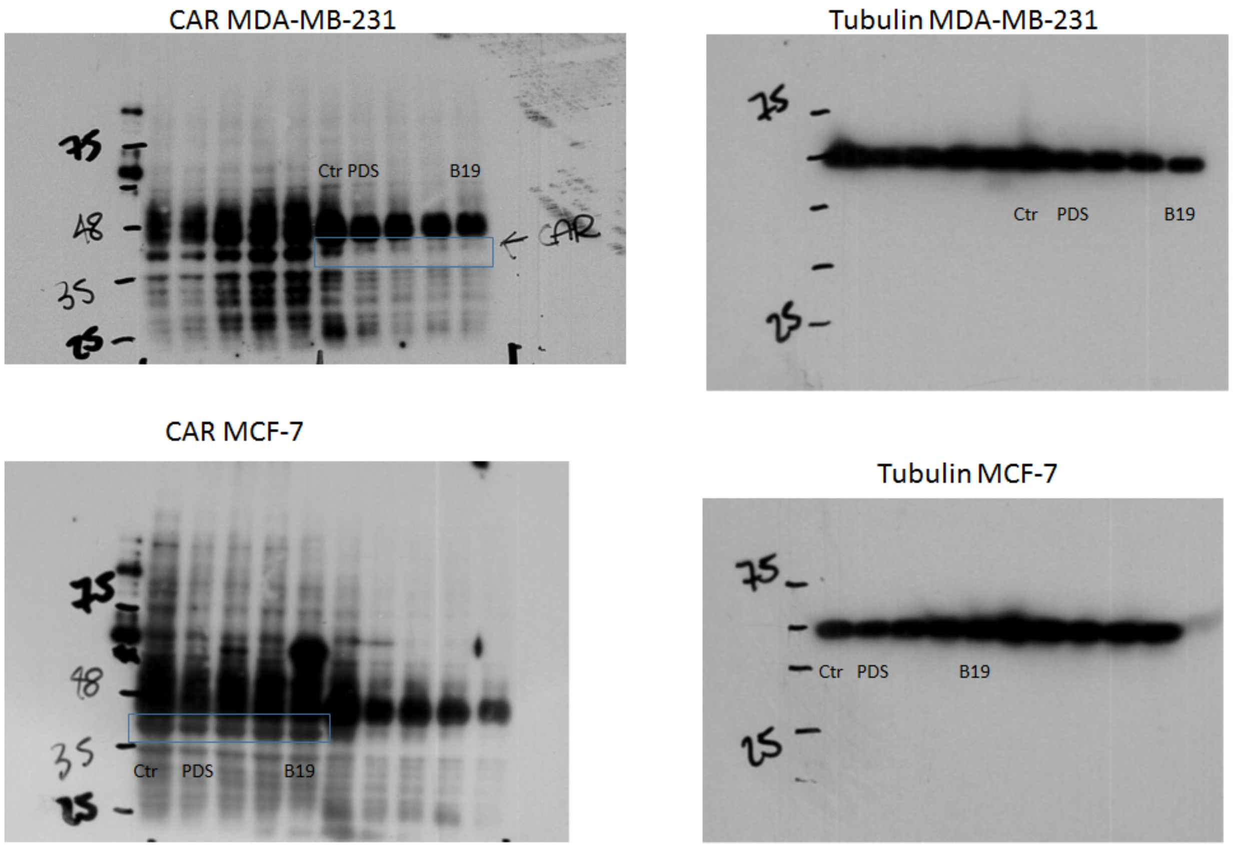

Figure S5 Original blot used for Figure 3E,F

The blots for CAR and α-tubulin are reported for MDA-MB-231 and MCF-7 cells. The bands of CAR expression are evidenced by blue rectangles and controls (CTR) and treatments (PDS and B19 at IC50) are indicated.

A

STING TUBULIN

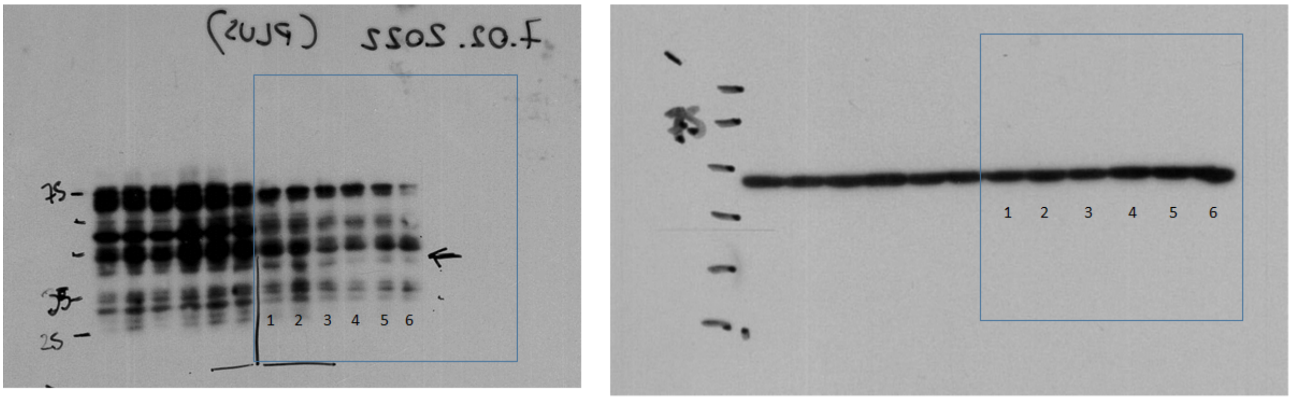

B

STING TUBULIN

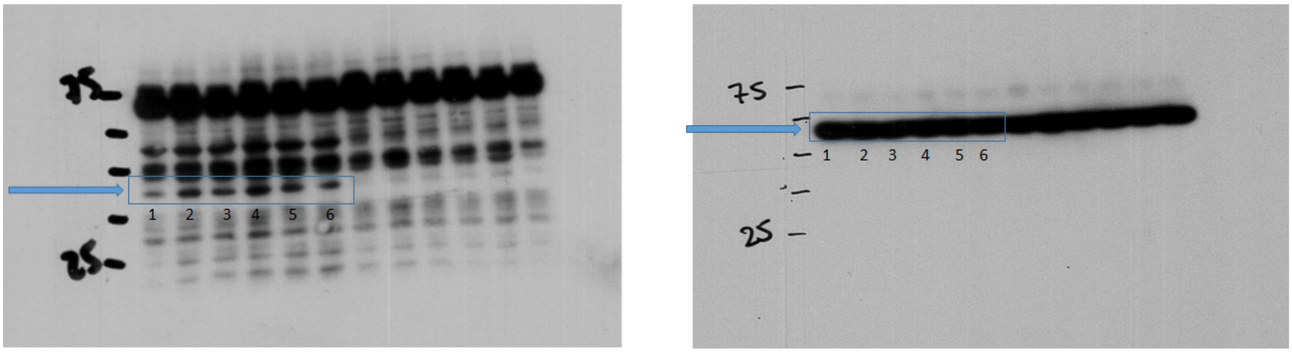

Figure S6 Original blot used for Figure 6

The blots for STING and tubulin are reported for MDA-MB-231 cells (A) and MCF-7 cells (B). The bands (evidenced by blue rectangles) are numbered and represent: lane 1= control untreated cells, lane 2= treatment with dl922-947, lane 3= B19, lane 4= PDS, lane 5= dl922-947+B19, lane 6= dl922-947+PDS. We have to highlight as already reported for other STING antibodies used in literature that, although the antibody was purchased and freshly used, it gave problems of un-specificity.
